# Beetroot (*Beta vulgaris*) Extract against *Salmonella* Typhimurium via Apoptosis-Like Death and Its Potential for Application in Cooked Pork

**DOI:** 10.3390/ijms241814217

**Published:** 2023-09-18

**Authors:** Shaoying Gong, Chaoqin Jiao, Ling Guo, Yujun Jiang

**Affiliations:** Key Laboratory of Dairy Science, Ministry of Education, College of Food Science, Northeast Agricultural University, Harbin 150030, China; gong_shaoying@126.com (S.G.); jiaochaoqin@126.com (C.J.)

**Keywords:** *S.* Typhimurium, natural extract, antibacterial activity, apoptosis-like death, ROS, cooked pork

## Abstract

*Salmonella* Typhimurium is a common foodborne pathogen in meat and meat products, causing significant harm and losses to producers and consumers. The aim of this study was to investigate the antibacterial activity and possible mechanisms of beetroot (*Beta vulgaris*) extract against *S.* Typhimurium, as well as the application potential in cooked pork. The results suggested beetroot extract could inhibit *S.* Typhimurium with a minimum inhibitory concentration (MIC) of 20 mg/mL. After treatment with beetroot extract (1 or 2 MIC), *S.* Typhimurium exhibited the characteristics of apoptotic-like death (ALD), such as membrane depolarization, phosphatidylserine (PS) externalization, caspase-like protein activation, and DNA fragmentation. Further research has shown that the ALD induced by beetroot extract in *S.* Typhimurium was caused by reactive oxygen species (ROS) consumption, which was different from most natural products. The treatment of cooked pork with beetroot extract could reduce the number of *S.* Typhimurium, lower pH, defer lipid oxidation, and improve the colour. These results indicate that beetroot extract can inhibit *S.* Typhimurium through the ALD mechanism and has potential as an antibacterial agent against *S.* Typhimurium in ready-to-eat meat products.

## 1. Introduction

Ready-to-eat (RTE) meat products are favoured by consumers for their taste, nutritional value, and convenience of consumption; however, RTE meat products are highly susceptible to bacterial contamination during production, processing, and transportation, leading to the occurrence of foodborne diseases [1,2]. *S.* Typhimurium is a Gram-negative bacterium that causes gastroenteritis, bacteraemia, typhoid fever, and focal infection and is one of the main foodborne pathogens in pork and its products [3,4]. Pork products polluted with *S.* Typhimurium could lead to a change in colour, peculiar smell, and the development of amine [5]. Consequently, many pork industries face the problem of effectively inactivating *S.* Typhimurium to ensure the quality of pork products [4]. Lipid oxidation is another critical factor in the spoilage of meat, notably pork, which is highly perishable because it contains more unsaturated fatty acids than other meat [6]. The negative influence of lipid oxidation on the quality of the meat includes changes in colour, rancid odour, reduced shelf life, and toxic compound accumulation [7]. Synthetic preservatives, for instance, butylated hydroxyanisole and propyl gallate, can restrain the growth of *S.* Typhimurium and lipid oxidation in meat; however, consumers have a negative perception regarding their possible toxicological and adverse health effects [7,8]. Therefore, food manufacturers are considering the use of plant extracts, such as cranberry anthocyanin, thyme, blueberry, and purslane extracts, to replace synthetic preservatives for restraining the growth of bacteria and for commanding the lipid oxidation course in RTE meat in China [6,8,9,10].

Beetroot (*Beta vulgaris*) is a root vegetable containing polyphenols, ascorbic acid, betaine, and flavonoids [11]. Beetroot extract is used as a food colourant or an additive in diverse food products, for instance, yoghurts and ice creams [12]. In addition, beetroot extract is considered a good source of compounds that exhibit antioxidant activities [12]. Research has also verified that beetroot extract can inhibit foodborne pathogens such as *Bacillus cereus*, *Escherichia coli*, *Listeria monocytogenes*, and *Cronobacter sakazakii* [11,12,13]; however, the antibacterial capacity of beetroot extract to inhibit *S.* Typhimurium and the underlying mechanism are not fully understood. In addition, there is little information regarding its antimicrobial and antioxidant effects in pork.

Apoptosis is genetically controlled programmed cell death that generally occurs in eukaryotic cells, and has the advantage of occurring without releasing harmful substances into the body [14]. A cell death mechanism similar to cell apoptosis has been discovered in bacteria and named apoptosis-like death (ALD) [15]. As far as we know, natural products such as curcumin, chlorogenic acid, coprisin, apigenin, indole-3-carbinol, silibinin, naringin, isoquercitrin, pterostilbene, *Sonchus brachyotus* DC, *Hypericum* extract, and *Portulaca oleracea* L. flavonoid extract can cause bacterial ALD [16,17,18,19,20,21,22,23,24,25,26,27]. The antibacterial mechanism of these substances through ALD are concentrated in *E. coli*, *Staphylococcus aureus*, *Micrococcus luteus*, and *B. cereus*. At present, there are no studies reporting ALD in *S.* Typhimurium. In addition, our previous research indicated that beetroot extract can act against *L. monocytogenes*, *B. cereus*, and *C. sakazakii* through the ALD mechanism [11,13,28]; however, it is unclear whether beetroot extract can inhibit *S.* Typhimurium through ALD. Therefore, we investigated whether beetroot extract can inhibit *S.* Typhimurium through ALD and evaluated the possibility of using beetroot extract as an antibacterial agent in cooked pork. Our results confirmed that beetroot extract could inhibit *S.* Typhimurium through the ALD mechanism and has the potential for application in cooked pork.

## 2. Results and Discussion

### 2.1. Minimum Inhibitory Concentration (MIC) and Time–Kill Kinetics Assay

**Figure 1:**
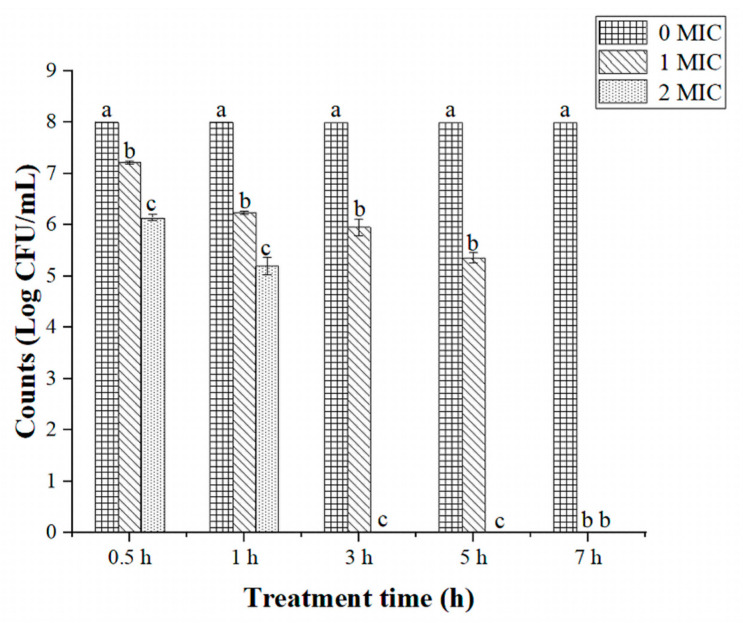
Time-kill kinetics of *Salmonella* Typhimurium after treatment with different concentrations of beetroot extract (0, 1, or 2 MIC). Detection limit of <10 CFU/mL. Values represent the means of independent triplicate measurements. Error bars denote standard error. Different letters denote significant differences between treatments within the same incubation time-points (*p* < 0.05).

Many studies have reported the antibacterial effects of natural plant extracts on *S.* Typhimurium [29,30]. In this study, we found that the MIC of beetroot extract inhibiting *S.* Typhimurium was 20 mg/mL. A study has revealed that *Chrysanthemum* buds’ crude extract could inhibit *S.* Typhimurium, with the MIC being 40 mg/mL [29]. The MIC of *Amaranthus spinosus* extract to inhibit *S.* Typhimurium has been reported as 129 mg/mL [31]. Compared with other natural products, beetroot extract showed better inhibition activity towards *S.* Typhimurium (MIC = 20 mg/mL). The antibacterial activity of beetroot extract may be related to its rich content of flavonoids, alkaloids, polyphenols, and organic acids, which have been reported to exhibit strong antibacterial ability [11,12]. In addition, the ability of beetroot extract to inhibit *S.* Typhimurium was evaluated through the time–kill kinetics assay (Figure 1). The growth of *S.* Typhimurium (10^8^ log CFU/mL) was totally inhibited (detection limit of <10 CFU/mL) after treatment with 1 MIC (20 mg/mL) and 2 MIC (40 mg/mL) of beetroot extract for 7 and 3 h, respectively; therefore, beetroot extract exerts effective antibacterial effects that inhibit *S.* Typhimurium.

### 2.2. Effects of Beetroot Extract on ALD of S. Typhimurium

**Figure 2:**
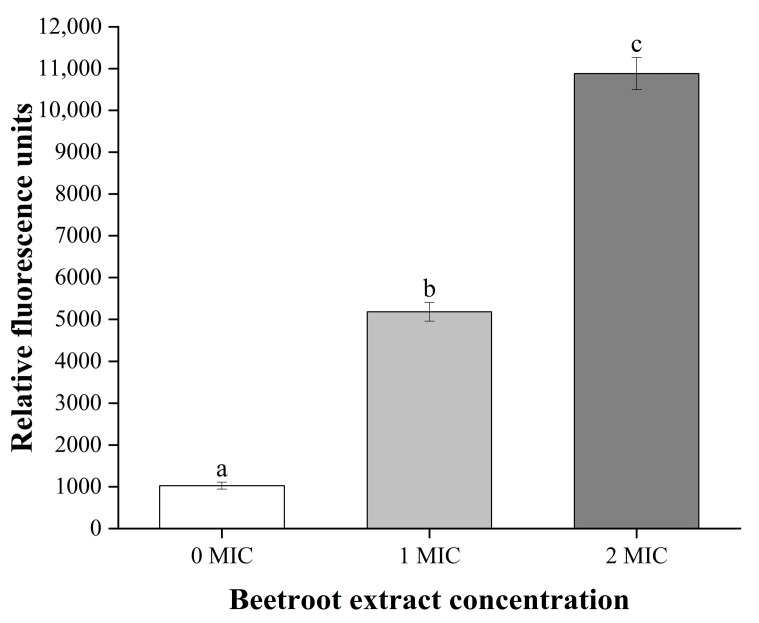
Change in membrane potential of *Salmonella* Typhimurium after treatment with 0, 1, or 2 MIC of beetroot extract. Values represent the means of independent triplicate measurements. Error bars represent the standard error. Different letters denote significant differences (*p* < 0.05).

**Figure 3:**
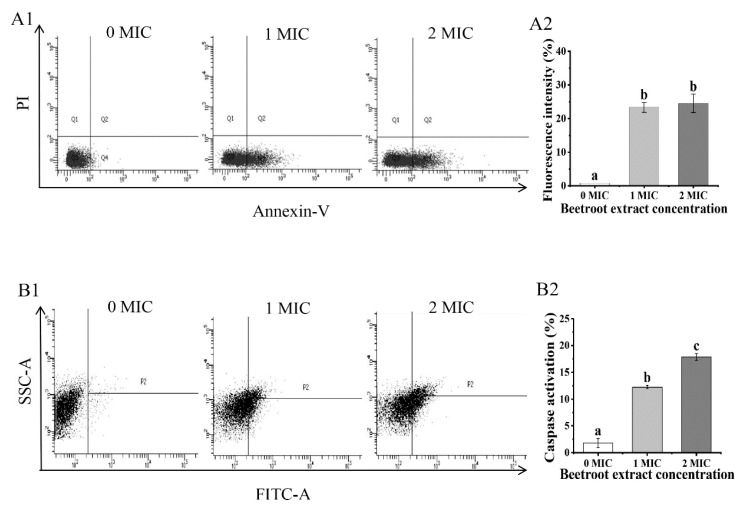

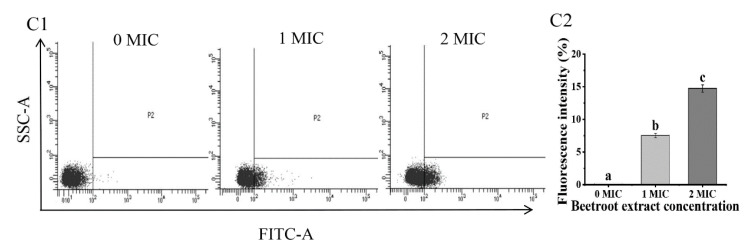
Change in PS externalization (**A1**,**A2**), caspase-like protein activation (**B1**,**B2**), and DNA fragmentation (**C1**,**C2**) of *Salmonella* Typhimurium after treatment with 0, 1, or 2 MIC of beetroot extract. Values represent the means of independent triplicate measurements. Error bars denote standard error. Different letters denote significant differences (*p* < 0.05).

To investigate whether beetroot extract can inhibit *S.* Typhimurium through the ALD mechanism, we evaluated the characteristics of ALD, which include membrane potential, phosphatidylserine (PS) externalization, caspases-like protein activation, and DNA fragmentation [26]. The DiBAC4(3) fluorescence-stained *S.* Typhimurium treated with 1 and 2 MIC of beetroot extract was markedly enhanced (*p* < 0.05) compared with the control (0 MIC), suggesting that *S.* Typhimurium treated with beetroot extract displayed membrane depolarization (Figure 2). PS, a phospholipid component, is present in the inner lobules of the plasma membrane. When cells undergo apoptosis, PS is no longer limited to the cytoplasmic side, resulting in PS externalization [21]. Therefore, we evaluated whether beetroot extract can cause PS translocation of *S.* Typhimurium. As shown in Figure 3(A1,A2), the apoptosis rate (lower right quadrant) of *S.* Typhimurium markedly increased after treatment with beetroot extract compared with the control (*p* < 0.05). Our results indicate that beetroot extract can result in *S.* Typhimurium PS externalization and suggest that *S.* Typhimurium may have undergone ALD. To further confirm that beetroot extract induces *S.* Typhimurium death through ALD, we evaluated the activity of caspase proteins, which are one of the key factors regulating ALD. Caspase is an inactive zymogen that, when appropriately stimulated, can activate target proteins and other caspases, leading to ALD [32]. In this study, the fluorescence intensity was markedly enhanced in *S.* Typhimurium treated with beetroot extract (*p* < 0.05) compared with the control (Figure 3(B1,B2)). This indicates that beetroot extract can activate caspase-like proteins in *S.* Typhimurium. In apoptotic cells, DNA breaks into small-molecular-weight fragments, which is a marker of late apoptosis [17]. TUNEL-positive signals were markedly enhanced (*p* < 0.05) in a dose-reliant pattern in *S.* Typhimurium treated with beetroot extract (Figure 3(C1,C2)). Almost no TUNEL-positive cells were measured in cells not treated with beetroot extract. These results imply that beetroot extract could cause *S.* Typhimurium DNA fragmentation. In summary, beetroot extract can inhibit *S.* Typhimurium through the ALD mechanism. Similarly, Kim, Woo, and Lee [19] found that apigenin can induce these characteristics of ALD in *E. coli*, for instance, membrane depolarization, PS externalization, caspases-like protein activation, and DNA fragmentation. A similar phenomenon was found in genistein-induced ALD in *E. coli* [33]. To our knowledge, this is the first study to discover ALD in *S.* Typhimurium.

### 2.3. Intracellular Reactive Oxygen Species (ROS) Level

**Figure 4:**
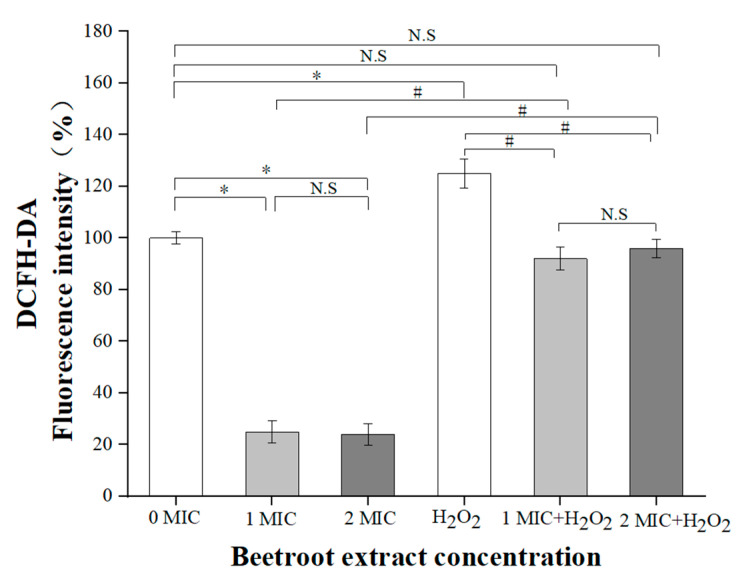
Intracellular ROS level of *Salmonella* Typhimurium after treatment with 0, 1, or 2 MIC of beetroot extract. Values represent the means of independent triplicate measurements. Error bars denote standard deviation. (N.S: not significant; * *p* < 0.05 compared to control group; # *p* < 0.05 compared to H_2_O_2_ treated sample).

According to reports, antibiotics can induce bacterial ALD by interacting with bacterial targets to produce excessive ROS [33,34]. Beetroot is well-known for its strong antioxidant properties [12]; thus, we assessed the ROS level in *S.* Typhimurium after treatment with beetroot extract. As shown in Figure 4, compared with the control, the ROS level of *S.* Typhimurium treated with beetroot extract was significantly reduced (*p <* 0.05). Moreover, the difference in the ROS level in *S.* Typhimurium between 1 and 2 MIC of beetroot extract treatment was not significant (*p* > 0.05). This indicates that beetroot-extract-induced *S.* Typhimurium ALD is not related to ROS overproduction, which is different from most natural-product-induced bacterial ALD, such as apigenin, curcumin, ethanol extracts from *Sonchus brachyotus* DC, etc. [16,25,33]. Some studies reported that the depletion of ROS can affect signalling pathways and even lead to cell death [35,36]. In addition, one study has suggested that ROS consumption could induce ALD in *E. coli* [21]. To verify that the ALD of *S.* Typhimurium induced by beetroot extract is caused by ROS consumption, we treated *S.* Typhimurium with beetroot extract and hydrogen peroxide (H_2_O_2_). The results showed that after co-treatment with beetroot extract and H_2_O_2_, the ROS consumption of *S.* Typhimurium was alleviated, and the level was comparable to the control. In addition, the ROS level of *S.* Typhimurium increased after treatment with H_2_O_2_ only. This confirms that the ALD induced by beetroot extract in *S.* Typhimurium is induced by ROS consumption. A similar situation has been reported in chlorogenic-acid-induced ALD in *E. coli* [17]. These results illustrate that the ROS level is a main regulator of ALD in *S.* Typhimurium treated with beetroot extract. This study is the first to discover that *S.* Typhimurium ALD can be induced through ROS consumption.

### 2.4. Antibacterial Effects of Beetroot Extract in Cooked Pork

**Figure 5:**
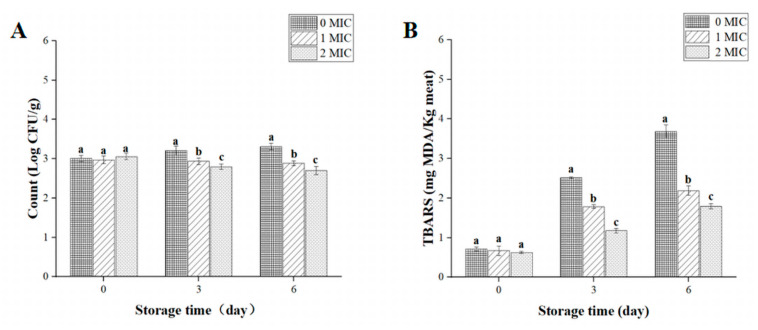
*Salmonella* Typhimurium counts (**A**) and TBARS values (**B**) of cooked pork during storage at 4 °C following treatment with 0, 1, or 2 MIC of beetroot extract. Values represent the means of independent triplicate measurements. Error bars denote standard error. Different letters indicate significant differences between treatments within the same incubation time point (*p* < 0.05).

The growth-inhibiting influences of beetroot extract on *S.* Typhimurium inoculated in cooked pork are depicted in Figure 5A. Compared with the control, the count of *S.* Typhimurium markedly reduced (*p* < 0.05) in cooked pork treated with beetroot extract; furthermore, the bacterial count decreased with an increase in the treatment concentrations of beetroot extract during storage.

As natural antimicrobials and natural antioxidants, natural plant extracts have the potential to enhance the entire quality of meat products [8,37,38]. Cordeuk with concentrations of 0.5–1.5% could result in 0.35–1.0 log CFU/g reductions in *S.* Typhimurium in Turkish-style meatballs [39]. Similarly, Xing et al. [29] found that 80 mg/mL of *Chrysanthemum* buds’ crude extract could reduce approximately 1-log CFU/g of *S.* Typhimurium in cooked chicken. We found that treatment with beetroot extract (2 MIC) reduced the *S.* Typhimurium count (about 0.61 log CFU/g) in cooked pork. Our research demonstrated that the application of beetroot extract could inhibit *S.* Typhimurium in cooked pork.

### 2.5. pH Value

The pH value of cooked pork treated with beetroot extract (1 or 2 MIC) was markedly decreased in comparison to the control samples (*p* < 0.05) during storage (Table 1); moreover, the pH value of the samples with the same treatment did not change significantly within 6 days (*p* > 0.05). Studies have shown that the addition of natural antimicrobials and antioxidants could reduce the pH values of meat products [40,41]. Our findings are analogous to those of Gong et al. [8], who verified that the addition of cranberry anthocyanin reduced the pH value of cooked pork. Similarly, the addition of *Amaranthus tricolor* crude extract reduced the pH value of cooked pork during 6 days of storage [42]. We speculate that the decrease in the pH value of cooked pork may be related to the rich organic acid and other acidic components in the beetroot extract, and on the other hand, it may be related to microbial metabolism.

### 2.6. Lipid Oxidation

There are two primary factors that determine the quality loss and shortened guarantee period of pork. The first factor is bacterial pollution, and the second factor is lipid oxidation [37]. Accordingly, inhibiting lipid oxidation is crucial for meat storage. In our study, the TBARS value of the cooked pork samples treated with 1 and 2 MIC of beetroot extract was markedly lower (*p* < 0.05) than the control (Figure 5B). In addition, the TBARS value of the cooked pork reduced as the concentration of the beetroot extract increased during storage. These results verified that beetroot extract can efficaciously reduce the TBARS value of cooked pork during storage, which may be related to the antioxidant and antibacterial properties of the polyphenols and flavonoids in the beetroot extract. Research has suggested that the addition of *Ephedra alata* extract could reduce the TBARS values of beef, manifesting that the variation in the TBARS values were connected with the antioxidant and antibacterial activities of the extract [43]. Similarly, adding an extract of *Morus alba* L. leaves could reduce the TBARS value of chilled pork and effectively maintain the quality of the pork [44].

### 2.7. Colour

Colour is important in meat as it is an indicator for appraising its freshness, quality, and shelf life [45]. In this study, compared with the control, the addition of beetroot extract reduced the L* and b* values and increased (*p* < 0.05) the a* and ΔE* values of all of the cooked pork samples during the same storage period (Table 2), and the change in colour is dose-dependent. The colour change in the samples may be attributed to the red pigment in beetroot extract. Likewise, the addition of chokeberry extract also could reduce the L* and b* values and augment the a* values of pork products, suggesting the change in the colour is relational with the colour of the extract [46]. Moreover, the addition of cranberry anthocyanins has been shown to increase the ΔE* value of cooked pork and cooked beef samples, similar to the present findings [8]. In addition, one study has shown that meat products with red and a cured colour compared to brown are more beneficial for attracting consumers [47]. Therefore, the treatment of cooked meat with beetroot extract is expected to become a suitable alternative to achieve the flesh colour desired by consumers.

## 3. Materials and Methods

### 3.1. Beetroot Extract and Bacterial Strain

The beetroot extract used in this present research was bought from Xi’an Ruiying Biological Technology Co., Ltd. (Xi’an, China). The chemical composition, as shown in our previous research based on type and proportion, consisted of flavonoids (16.64%), alkaloids (12.21%), polyphenols (11.01%), terpenoids (11.01%), and organic acids and derivatives (6.58%) as the five dominant compounds [11]. *S.* Typhimurium ATCC 14028 was provided by the American Type Culture Collection (ATCC, Manassas, VA, USA). *S.* Typhimurium was stored in centrifuge tubes (containing 80% glycerol) and frozen in a refrigerator. In total, 300 µL bacterial solution was shifted to Luria–Bertani broth and cultivated on a shaker at 37 °C for 24 h.

### 3.2. Determination of MIC

The MIC of the beetroot extract against *S.* Typhimurium was determined using the agar dilution method [8]. The *S.* Typhimurium (10^4^ CFU/mL) were treated with diverse concentrations of beetroot extract (0, 2.5, 5, 10, 20, 40, or 80 mg/mL). Next, 2 μL of the bacterial culture was inoculated on tryptic soy agar (TSA) and cultivated in an incubator at 37 °C for 24 h. The lowest concentration of beetroot extract in which *S.* Typhimurium could not be detected was considered the MIC.

### 3.3. Time–Kill Kinetics Assay

*S.* Typhimurium (10^8^ CFU/mL) was treated with beetroot extract (1 or 2 MIC) for 0.5, 1, 3, 5, and 7 h at 37 °C, and cultures untreated with beetroot extract served as the control [17]. Next, the bacterial suspension (100 µL) was added onto TSA and cultured for 24 h at 37 °C to calculate the survival populations.

### 3.4. Membrane Potential Assessment

The membrane potential of *S.* Typhimurium was determined utilising a Bis-(1,3-dibutylbarbituric acid) trimethine oxonol [DiBAC4(3)] probe (Beijing Solarbio Sciences and Technology Co., Ltd., Beijing, China) [29]. Briefly, *S.* Typhimurium (10^8^ CFU/mL) was shifted to 96-well plates (black), and the DiBAC4(3) reagent was added. After cultivation for 0.5 h, beetroot extract (0, 1, or 2 MIC) was added. Lastly, the fluorescence value of *S.* Typhimurium was detected using a microplate reader with emission and excitation wavelengths of 515 and 492 nm, respectively (Infinite 200 PRO, Tecan, Grodlg, Austria).

### 3.5. PS Externalization of S. Typhimurium

The PS externalization of *S.* Typhimurium was assessed using Annexin V-FITC/PI (Beijing Solarbio Sciences and Technology Co., Ltd., Beijing, China) [37]. *S.* Typhimurium (10^8^ CFU/mL) were treated with 0, 1, or 2 MIC of beetroot extract and cultivated at 37 °C for 3 h. After being washed with PBS and centrifuged, *S.* Typhimurium was re-dissolved in a binding buffer. Annexin V-FITC reagent (5 μL) was added to the culture and cultivated in the dark for 10 min. Next, PI reagent (5 μL) was added to the culture, and then the FACSVerse flow cytometer was utilised to detect the fluorescence value of the sample (Becton Dickinson, Franklin Lakes, NJ, USA).

### 3.6. Caspase-Like Protein Expression of S. Typhimurium

CaspACE FITC-VAD-FMK In Situ Marker (Promega, Madison, WI, USA) was used to detect whether the beetroot extract could activate the caspase-like protein of *S.* Typhimurium [17]. *S.* Typhimurium (10^8^ CFU/mL) was treated with beetroot extract (0, 1, or 2 MIC) at 37 °C for 3 h. Afterwards, the culture was centrifuged to collect *S.* Typhimurium, and the FITC-VAD-FMK reagent (5 μmol/L) was added. After 1 h, the *S.* Typhimurium was gathered and redissolved in PBS and detected using a FZCSARIA flow cytometer.

### 3.7. DNA Fragmentation of S. Typhimurium

The DNA fragmentation of *S.* Typhimurium was assessed using a TUNEL Apoptosis Assay Kit (Beijing Solarbio Sciences and Technology Co., Ltd.) [27]. *S.* Typhimurium (10^8^ CFU/mL) was treated with beetroot extract (0, 1, or 2 MIC) and incubated at 37 °C for 3 h. After being washed and centrifuged, *S.* Typhimurium was fixed with 4% paraformaldehyde for 1.5 h. Then, *S.* Typhimurium was redissolved in PBS (with 0.3% Triton X-100) and incubated for 5 min (25 °C). Next, 5 μL of TUNEL detection reagent was added and incubated in the dark for 1 h. Finally, the fluorescence value of the sample was detected utilising a FACSVerse flow cytometer.

### 3.8. Estimate of ROS Level of S. Typhimurium

An ROS detection kit (S0033; Beyotime Bioengineering Institute, Shanghai, China) was used to measure the intracellular ROS level of *S.* Typhimurium according to the manufacturer’s instructions [20]. *S.* Typhimurium (10^8^ CFU/mL) was co-incubated with DCFH-DA for 20 min, and then beetroot extract was added and incubated at 37 °C for 3 h. The fluorescence intensity of the sample was measured using a microplate reader at an excitation wavelength of 488 nm and an emission wavelength of 525 nm.

### 3.9. Recovery of Intracellular ROS Depletion

According to the method of Lee and Lee [20], H_2_O_2_ was used to restore the ROS level of *S.* Typhimurium. *S.* Typhimurium was treated with H_2_O_2_ and beetroot extract, or H_2_O_2_ alone, and incubated at 37 °C for 3 h. Then, the intracellular ROS level was measured.

### 3.10. Preparation of Cooked Pork Samples

Fresh pork was provided by a supermarket in Harbin and stored in the laboratory fridge (4 °C). We purchased a total of 15 kg of fresh lean pork, which was purchased three times (about 5 kg each time). Every test was performed in triplicate. The pork was cut into blocks (about 15 g/block) and then put into a glass container for autoclaving for 15 min (121 °C). Each block of pork lost approximately 5 g, resulting in about 10 g of cooked pork samples.

### 3.11. Evaluation of S. Typhimurium in Cooked Pork

The antibacterial effect of beetroot extract against *S.* Typhimurium in cooked pork was assessed according to previous research [8]. The samples were divided into three equal parts using sterile tweezers, and then immersed in 0, 1, or 2 MIC of beetroot extract for 10 s, respectively. Then, the cooked pork was deposited in an aseptic glass container with sterile tweezers and left until the beetroot extract solution had been absorbed. *S.* Typhimurium (3 log CFU/g) was inoculated in the samples and then cultured in sterile bags (a sterile bag contained one cooked pork sample) for 6 days during storage (4 °C). The counts of *S.* Typhimurium were determined every 3 days during storage.

### 3.12. pH Value

The pH value of the cooked pork was evaluated using a pH meter (ST2100/F, OHAUS instruments Co., Ltd., Changzhou, Jiangsu Province, China ) [42]. The sample was mixed with 90 mL of deionised water and then measured utilising pH meter at 0, 3, and 6 days (4 °C).

### 3.13. Assessment of Lipid Oxidation

Adopting the approach described by Fan et al. [10], we measured changes in the TBARS values for samples treated with diverse concentrations of beetroot extract. Briefly, 10 g of cooked meat was added to 50 mL of 7.5% cold trichloroacetic acid (including 0.1% EDTA) for homogenisation for 0.5 h and then filtered. The supernatant was mixed with an equal amount of 2-thiobarbituric acid for 40 min (100 °C). Next, the mixture was centrifuged for 5 min (8000× *g*) and left to cool for 1 h at ambient temperature. Then, the supernatant was taken from the mixture and mixed with 5 mL of chloroform. Lastly, the absorbance of the sample was detected utilising a visible spectrophotometer. The wavelengths required to set the instrument were 532 and 600 nm, respectively. The TBARS values of the sample were determined during storage periods of 0, 3, and 6 days, respectively.

### 3.14. Assessment of Colour

The colour of the cooked pork samples was detected utilising a colourimeter (Cr-310, Minolta Co., Ltd. Shanghai, China) with an 8 mm aperture, based on a D65 illuminant and a 0° standard observer [31]. The colourimeter was calibrated using a normative white tile before measuring the samples. The colour of the cooked pork samples was detected at 0, 3, and 6 days (4 °C). Each slice of cooked pork underwent six measurements in diverse locations. The difference in the overall colour between the untreated samples and the samples treated with 1 and 2 MIC of beetroot extract was calculated utilising the following formula: ΔE* = [(L* _CONT_ – L* _N_)^2^ + (a* _CONT_ − a* _N_)^2^ + (b* _CONT_ − b* _N_)^2^]^1/2^ (*n* = 1, 2).

### 3.15. Statistical Analysis

The experiment was repeated three times with three parallel samples for each sample. All experimental data were analyzed using SPSS 25 software (SPSS, Inc., Chicago, IL, USA). We tested for significant differences between the means of the experimental data using Tukey’s multiple comparisons test. The difference between treatments was considered significant at *p* < 0.05. The experimental results were expressed as mean values with standard errors of the mean.

## 4. Conclusions

In this research, we demonstrated that beetroot extract can inhibit *S.* Typhimurium through ALD, which is caused by the consumption of ROS. In addition, beetroot extract can inhibit the growth of *S.* Typhimurium, reduce pH and TBARS values, and improve the colour of cooked pork during storage. These results illustrate that beetroot extract has good potential for use as a natural preservative to reduce the risk of *S.* Typhimurium contamination in cooked pork. This research broadens the perception of natural products as foodborne pathogen inhibitors and provides a theoretical basis for the mechanism of ALD.

## Figures and Tables

**Table 1:** pH values of cooked pork during storage at 4 °C following treatment with different concentrations of beetroot extract.

Storage Time (Day)	Treatments (Cooked Pork + Beetroot Extract)		
	Control	1 MIC	2 MIC
0	5.93 ± 0.01 ^a,A^	5.88 ± 0.01 ^b,A^	5.86 ± 0.01 ^b,A^
3	5.94 ± 0.01 ^a,A^	5.89 ± 0.00 ^b,A^	5.86 ± 0.01 ^c,A^
6	5.93 ± 0.01 ^a,A^	5.88 ± 0.01 ^b,A^	5.85 ± 0.01 ^c,A^

Values are mean ± standard error (SE) of triplicate determinations. Different lowercase letters indicate significant differences in meats treated with different concentrations of beetroot extract (*p* < 0.05). Different uppercase letters indicate significant differences due to the storage period (*p* < 0.05).

**Table 2:** Colour determination of cooked pork during storage at 4 °C following treatment with beetroot extract.

Storage Time (Day)	Treatments (Cooked Pork + Beetroot Extract)		
	Control	1 MIC	2 MIC
L*-value			
0	70.02 ± 0.07 ^a^	65.48 ± 0.17 ^b^	59.56 ± 0.11 ^c^
3	69.13 ± 0.36 ^a^	62.03 ± 0.16 ^b^	58.88 ± 0.04 ^c^
6	67.05 ± 0.38 ^a^	61.03 ± 0.47 ^b^	58.03 ± 0.02 ^c^
a*-value			
0	6.55 ± 0.16 ^a^	12.96 ± 0.23 ^b^	14.56 ± 0.10 ^c^
3	4.46 ± 0.02 ^a^	10.79 ± 0.03 ^b^	12.70 ± 0.04 ^c^
6	4.04 ± 0.02 ^a^	10.02 ± 0.01 ^b^	11.65 ± 0.06 ^c^
b*-value			
0	25.47 ± 0.17 ^a^	24.14 ± 0.11 ^b^	23.54 ± 0.16 ^c^
3	23.83 ± 0.03 ^a^	22.86 ± 0.06 ^b^	20.81 ± 0.04 ^c^
6	22.76 ± 0.07 ^a^	22.15 ± 0.01 ^b^	20.05 ± 0.09 ^c^
ΔE*-value			
0	—	7.98 ± 0.36 ^a^	13.33 ± 0.16 ^b^
3	—	9.57 ± 0.28 ^a^	13.50 ± 0.22 ^b^
6	—	8.54 ± 0.46 ^a^	12.10 ± 0.25 ^b^

L* = lightness, a* = redness, and b* = yellowness. Values are mean ± standard error (SE) of six determinations. Different lowercase letters represent significant differences at different concentrations (*p* < 0.05).

## Data Availability

The data in this study can be obtained by contacting the corresponding author.
